# Manipulation of Regulatory Dendritic Cells for Induction Transplantation Tolerance

**DOI:** 10.3389/fimmu.2020.582658

**Published:** 2020-10-14

**Authors:** Weitao Que, Wen-Zhi Guo, Xiao-Kang Li

**Affiliations:** ^1^Department of Hepatobiliary and Pancreatic Surgery, The First Affiliated Hospital of Zhengzhou University, Zhengzhou, China; ^2^Division of Transplantation Immunology, National Research Institute for Child Health and Development, Tokyo, Japan

**Keywords:** allograft, dendritic cell, T cell, tolerance, transplantation

## Abstract

Current organ transplantation therapy is life-saving but accompanied by well-recognized side effects due to post-transplantation systematic immunosuppressive treatment. Dendritic cells (DCs) are central instigators and regulators of transplantation immunity and are responsible for balancing allograft rejection and tolerance. They are derived from monocyte-macrophage DC progenitors originating in the bone marrow and are classified into different subsets based on their developmental, phenotypical, and functional criteria. Functionally, DCs instigate allograft immunity by presenting donor antigens to alloreactive T cells via direct, indirect, and semidirect recognition pathways and provide essential signaling for alloreactive T cell activation via costimulatory molecules and pro-inflammatory cytokines. Regulatory DCs (DCregs) are characterized by a relatively low expression of major histocompatibility complex, costimulatory molecules, and altered cytokine production and exert their regulatory function through T cell anergy, T cell deletion, and regulatory T cell induction. In rodent transplantation studies, DCreg-based therapy, by *in situ* targeting or infusion of *ex vivo* generated DCregs, exhibits promising potential as a natural, well-tolerated, organ-specific therapeutic strategy for promoting lasting organ-specific transplantation tolerance. Recent early-phase studies of DCregs have begun to examine the safety and efficacy of DCreg-induced allograft tolerance in living-donor renal or liver transplantations. The present review summarizes the basic characteristics, function, and translation of DCregs in transplantation tolerance induction.

## Introduction

Organ transplantation has progressed greatly over the past half-century to become the optimal treatment for a variety of end-stage organ diseases. However, the life-long, systemic immune suppression after a transplantation has major associated adverse effects, including severe infections, malignancies, psychosocial issues affecting patients, and a high cost to the health care system (1, 2). Immunosuppressive drugs can prevent acute cellular rejection but fail to control donor-specific antibody production and later-phase chronic organ rejection that may eventually lead to graft failure (3–5). Donor-specific tolerance is recognized as superior to systemic immunosuppression in being more conducive to donor organ acceptance without compromising host protective immunity. In this context, regulatory immune cell-based therapies are emerging as novel, promising strategies for establishing permanent donor-specific immune tolerance and minimizing or even obviating the need for immunosuppressive drugs after organ transplantation. Many types of regulatory immune cells, such as regulatory T cells (Tregs) (6), type 1 regulatory T cells (Tr1) (7), regulatory macrophages, regulatory B cells (Bregs) (5, 8), myeloid-derived suppressor cells (9), and regulatory dendritic cells (DCregs) (10), have already been investigated in animal transplantation models, and entered the clinical trials in organ transplantation and shown clear benefits in terms of safety and graft survival (11, 12). Among these cells, DCregs are particularly attractive due to their role as central regulators of the immune response.

## Basic Profile of DCs

DCs are a rare, heterogeneous population of the most efficient antigen-presenting cells (APCs) derived from bone marrow. They play a critical role in the instigation and regulation of the immune response (13, 14). DCs are distributed ubiquitously throughout the body and serve as immunologic sentinels specialized in sensing danger signals and capturing, processing, and presenting antigenic materials (13, 15–17). DCs can initiate both the innate and adaptive immune responses, for example, via natural killer (NK) cells and cytotoxic T cells. In these processes, DCs undergo complicated phenotypical and functional changes in response to the environment, signals, and antigens (18).

Based on their developmental, phenotypical, and functional features, DCs can be categorized into several different subsets. Traditionally, DCs were subdivided into classical or conventional DCs (cDCs), plasmacytoid DCs (pDCs), monocyte-derived DCs (MoDCs), and langerhans cells (LCs) (19). All subsets of DCs express major histocompatibility complex (MHC) class II and CD11c surface molecular markers. The cDC subtype regularly located in lymphoid organs and most non-lymphoid organs has a superior ability to capture, process, and present antigens to naïve T cells. Indeed, studies of transgenic mice, constitutive or conditionally deficient in cDCs, have confirmed the central role of cDCs in priming the naïve T cell response (20, 21). The cDC subtypes can be further subdivided into cDC1 (CD8α+/CD103+ in mice; CD141+ in humans) and cDC2 (CD11b+ in mice; CD1c+ in human) subsets. Moreover, the cDC1 subset is adept in cross-presentation and priming CD8+ cytotoxic T cells while the cDC2 subset is most proficient at driving the CD4+ T cell response (22–24). The pDCs patrolling the blood and peripheral organs excel in producing high levels of type I IFN in response to viruses and RNA/DNA or immune complexes, a direct consequence of their constitutively expressing IRF7 (25, 26). They also participate in antigen presentation, control the T cell response, and usually exhibit tolerogenic properties by favoring the generation of Tregs (27, 28). MoDCs, also known as inflammatory DCs, derive from monocytes infiltrating under inflammatory conditions and are capable of releasing large amounts of tumor necrosis factor alpha (TNF-α) and inducible nitric oxide synthase (iNOS) upon pathogen recognition (29). Although identified as a subset of DCs, MoDCs share many features with both cDCs and macrophages, and their classification is still debated (30, 31). LCs are a distinct subset of DCs resident in the epidermal layer of the skin. Although LCs are similar to cDCs in terms of phenotype and function, they also have unique differentiation and homeostatic features (19, 32). LCs are generated from embryonic hematopoietic precursors which are seeded in the skin in the prenatal period and self-renew *in situ* at a very low rate under physiological steady-state conditions without replenishment by blood-borne precursors (33, 34). In contrast to cDCs, LC development is independent of FMS-like tyrosine kinase 3(Flt3) and Flt3 ligand (Flt3L) but requires colony-stimulating factor 1 receptor (Csf-1R) like many tissue-resident macrophages, such as microglial cells and Kupffer cells (35, 36). Recently, IL-34 has been identified as the second functional ligand for Csf-1R and was required for the development of LCs and microglial cells (37). In the current classification of DCs, it is unclear whether DCregs constitute an independent DC subset or represent a specific functional state of DCs. In fact, most DC subsets can exert regulatory function through T cell anergy, T cell deletion, and Treg induction (38, 39).

**Figure 1:**
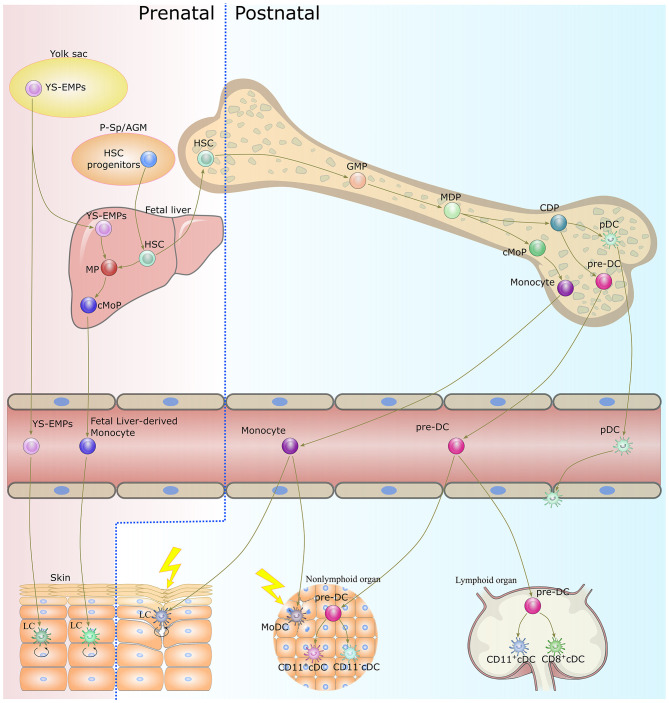
Origin and development of dendritic cells. With the exception of LCs, DCs develop from bone marrow-derived precursors. CDPs give rise to cDCs and pDCs. Monocytes differentiate into MoDCs in tissue as a consequence of inflammation or infection. LCs originate in prenatal precursor cells and are maintained locally by self-renewal under steady-state conditions. While under a severe inflammatory condition, LCs are replaced by blood-borne monocytes and acquire the capacity of self-renewal. DC, dendritic cell; LC, langerhans cells; CDP, common dendritic cell progenitor; cDC, classical dendritic cell; pDC, plasmacytoid dendritic cell; MoDC, monocyte-derived dendritic cell; YS-EMPs, Yolk sac-derived erythromyeloid progenitor cells; P-Sp/AGM para-aortic splanchnopleure/aorta, gonads, and mesonephros; HSC, hematopoietic stem cells; CMP, common myeloid progenitor cell; MP, myeloid progenitor cell; cMoP, common monocyte progenitor; GMP, granulocyte-macrophage progenitor; MDP, monocyte-macrophage DC progenitor.

The lifespan of DCs is generally short, and continuous replenishment from bone marrow progenitors is essential to maintaining DC homeostasis (40). Except for LCs, the majority of DC subsets originate from the same progenitors, namely monocyte-macrophage DC progenitors (MDPs), which reside in the bone marrow (19, 41) (Figure 1). MDPs further give rise to common monocyte progenitors (cMoPs) and common DC progenitors (CDPs) (42, 43). cMoPs develop into blood monocytes in the bone marrow but further differentiate into MoDCs in tissue as a consequence of inflammation or infection (29, 43–46). CDPs further give rise to pDCs and pre-DCs (47, 48). pDCs terminally differentiate into fully developed cells in the bone marrow, then migrate out to patrol the blood and peripheral organs (49, 50). Pre-DCs migrate out of the bone marrow through the blood to seed non-lymphoid and lymphoid organs, where they terminally differentiate into cDCs (36, 51, 52). LCs derive predominantly from embryonic fetal liver monocytes with a minor contribution from yolk sac-derived macrophages and are maintained locally by self-renewal under steady-state conditions (33, 53). In severe inflammatory conditions, LCs are replaced by blood-borne monocytes and acquire the capacity for self-renewal (35, 54).

## Function of DCs in Transplantation

DCs are critical to linking the innate and adaptive response in transplantation, in other words, to initiating robust, donor-specific, alloreactive T cell activation. During a classical immune response, immature DCs sense the presence of damage- and pathogen-associated molecular patterns (DAMPs and PAMPs), the so-called “Signal 0s,” from damaged cells and microbial molecules, respectively, via pattern recognition receptors (PRRs) (55, 56). These PRRs mediate internalized antigens and their routing to antigen-processing pathways (57). Subsequently, PRRs activate a series of intracellular pro-inflammatory molecular signaling cascades, such as interferon-responsive factor and nuclear factor kappa B pathways (58, 59). Activation of these signaling pathways leads to maturation of DCs, characterized by upregulation of MHC molecules, costimulatory molecules (e.g., CD80, CD86), chemokine receptors (e.g., C-C chemokine receptor type 7, CCR7), adhesion molecules (e.g., CD62L), and pro-inflammatory cytokines (e.g., TNF-α, IL-12) (60–62). Chemokine receptors and adhesion molecules permit DCs to migrate to lymphoid organs, where they contact and prime T cells (63–65). Antigens loaded on MHC class I molecules are presented to CD8+ T cells, whereas antigens loaded on MHC class II molecules are presented to CD4+ T cells. Costimulatory molecules and pro-inflammatory cytokines provide the essential signals for T cell activation (66, 67).

**Figure 2:**
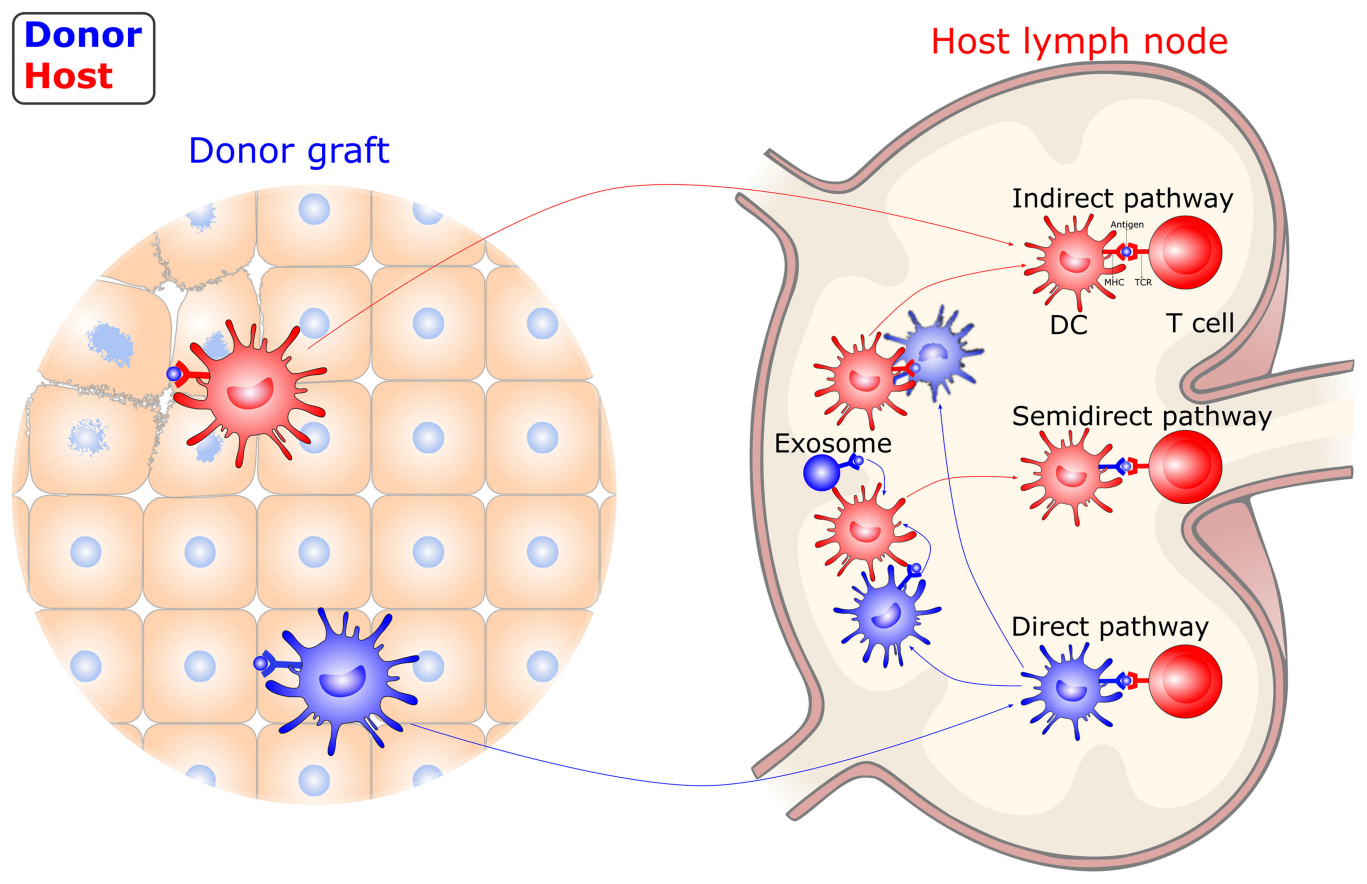
Allorecognition pathways in transplantation. DCs instigate allograft immunity by presenting donor antigens to alloreactive T cells via direct, indirect, and semidirect recognition pathways. In the direct pathway, donor-derived DCs directly present intact donor (allogeneic) MHC molecules to alloreactive T cells. In the indirect pathway, host-derived DCs present donor-derived antigens, captured from damaged graft tissue or dying, donor-derived DCs, to alloreactive T cells. In the semidirect pathway, intact donor MHC molecules transferred to host DCs by cell-cell contact or extracellular vesicles are recognized. DC, dendritic cell; MHC, major histocompatibility complex; TCR, T-cell receptor.

Unlike immune responses to conventional antigens, the trigger of allograft immunity relies on both donor- and host-derived DC-mediated antigen recognition (Figure 2). Following transplantation, donor-derived DCs migrate out of the graft to the graft-draining lymphoid tissues, where they directly present intact, donor (allogeneic) MHC molecules to alloreactive T cells. The direct allorecognition pathway is considered to dominate primary immune responses following allografting, which leads to acute graft rejection. The donor-derived DCs are rapidly eliminated by the early response of NK cells. Thus, direct allorecognition decreases along with a drop in the number of donor-derived DCs after transplantation. Meanwhile, host-derived DCs capture, process, and present donor-derived antigens to alloreactive T cells via the indirect pathway. These donor-derived antigens originate in damaged donor cells within a graft or in dying donor-derived DCs within draining secondary lymphoid organs. The indirect allorecognition pathway partially contributes to the early alloresponses and gradually dominates, leading to alloantibody production and chronic rejection. In addition, alloreactive T cells can also be stimulated via the semidirect allorecognition pathway, i.e., the recognition of intact donor MHC molecules transferred to host DCs by cell-cell contact or extracellular vesicles. The semidirect allorecognition pathway generates effector T cells that are donor MHC-restricted, as with the direct allorecognition pathway. Although each of these allorecognition pathways can solely or synergistically lead to allograft rejection, the maturity of DCs greatly influences the magnitude and quality of the T cell response (68–70).

**Figure 3:**
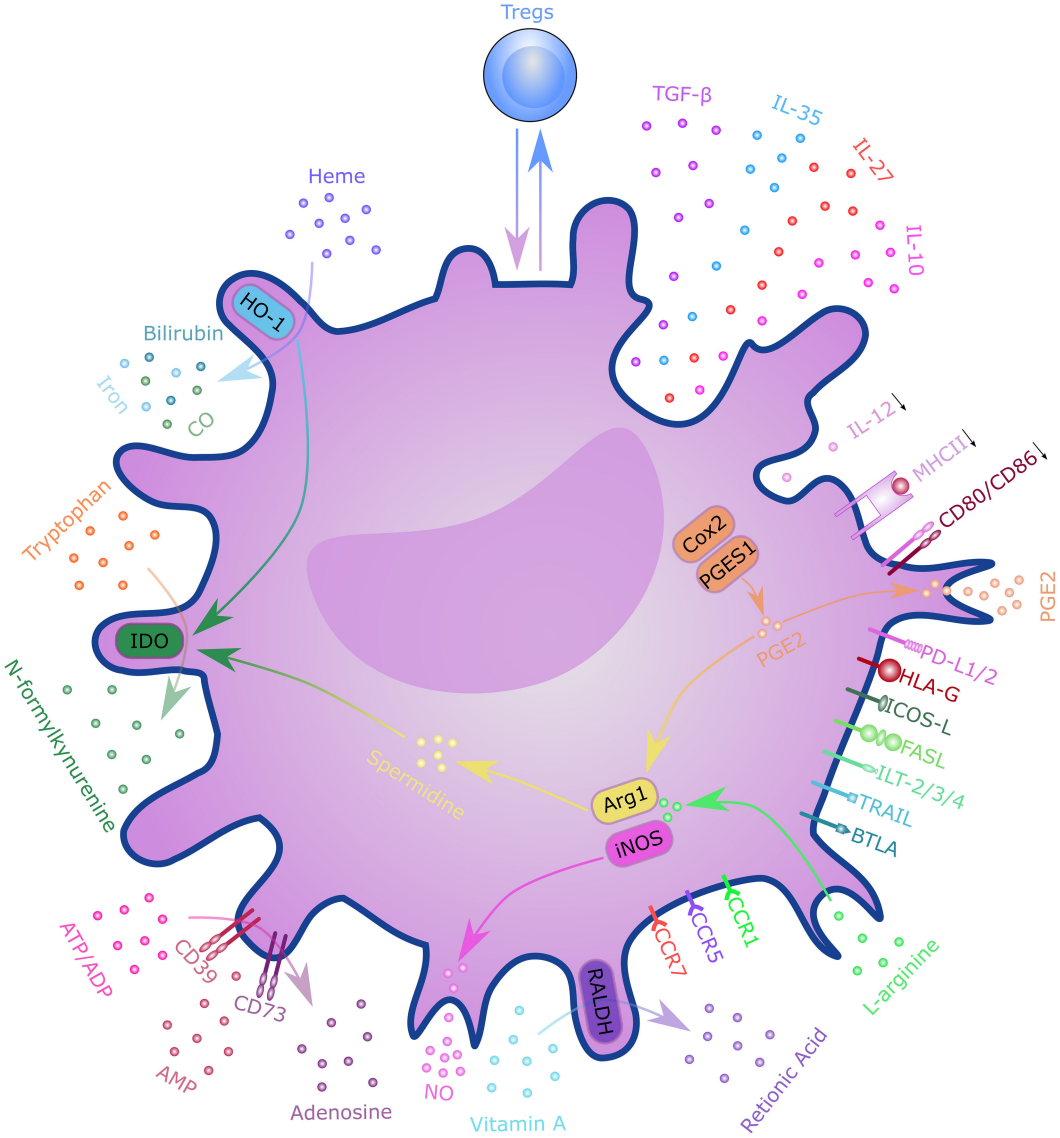
Mechanisms of regulatory dendritic cells. DCregs exert a regulatory function through T cell anergy, T cell deletion, and regulatory T cell induction. These mechanisms involve direct cell–cell interactions through surface molecules, as well as through the immunosuppressive milieu via secretory factors. DCregs and Tregs interact with each other through a self-maintaining regulatory loop. DC, dendritic cell; PD-L1/2, programmed cell death ligand 1; ICOS-L, inducible T-cell costimulatory ligand; TGF-β, transforming growth factor β; HLA-G, human leukocyte antigen G; FasL, Fas ligand; ILT-2/3/4, immunoglobulin-like transcript-2/3/4; TRAIL, tumor necrosis factor-related apoptosis inducing ligand; BTLA, B- and T-lymphocyte attenuator; CCR7, C-C chemokine receptor 7; CXCR-4, C-X-C chemokine receptor type 4; IL, interleukin; RALDH, retinaldehyde dehydrogenases; NO, nitric oxide; ATP, adenosine triphosphate; ADP, adenosine diphosphate; AMP, adenosine monophosphate; IDO, indoleamine 2,3-dioxygenase; CO, carbon monoxide; HO-1, Heme oxygenase 1; Cox2, cyclooxygenase 2; PGES1, prostaglandin E synthase-1; ARG1, arginase 1; iNOS, inducible nitric oxide synthase; MHCII, major histocompatibility complex class II; PGE2, Prostaglandin E2; Tregs, regulatory T cells.

T cells are critical players in transplantation immunity, which directly mediates allograft rejection. The activation of alloreactive T cells depends on three distinct DC-derived signals, including T-cell receptor engagement (signal 1), costimulation (signal 2), and cytokine stimulation (signal 3) (66). These DC-derived signals determine the fate of alloreactive T cells and tilt the balance toward graft survival or rejection (71). DCregs comprise a heterogeneous population of immature or semi-mature DCs characterized by a relatively low expression of MHC class II molecules, costimulatory molecules, and altered cytokine production. DCregs are thought to exert their regulatory function through various mechanisms (Figure 3). With the presentation of low levels of antigens in the absence of costimulatory molecules, such as CD80 and CD86, T cells will become anergic and lose their ability to proliferate (72, 73). The presence of coinhibitory signals during T cell activation, such as programmed cell death 1(PD-1) and PD ligand 1(PD-L1) interaction, CD80/CD86 and cytotoxic T lymphocyte antigen 4 (CTLA-4) interaction, inducible T-cell costimulator (ICOS) and ICOS ligand (ICOS-L) interaction, and heme oxygenase-1 (HO-1), can also lead to T cell anergy (74–78). HO-1 expression was also shown to be beneficial to DC survival and immunoregulatory properties (79, 80). Altered cytokine production; low levels of pro-inflammatory cytokines, such as IL-12; and high levels of anti-inflammatory cytokines, such as transforming growth factor β (TGF-β) and IL-10 mediate T cell anergy and Treg induction (81). DCregs can also induce clonal deletion of alloreactive T cells via the Fas/FasL pathway or indoleamine 2,3-dioxygenase (IDO)-induced apoptosis (82, 83). Furthermore, DCregs can induce several subtypes of regulatory lymphocytes, including classical Foxp3+ Tregs, Tr1, CD8+ Tregs, and Bregs via mechanisms involving direct cell-cell interaction signaling through surface molecules (e.g., immunoglobulin-like transcript (ILT)-3/4, CTLA-4, PD-L1, FasL, ICOS-L, and others), as well as the immunosuppressive milieu through secretory factors (e.g., IL-10, TGF-β, IL-27, IL-35, IDO, retinoic acid, adenosine, HO-1, and nitric oxide). DCregs and Tregs interact with each other through a self-maintaining regulatory loop required for maintaining immune tolerance (84).

## *Ex vivo* Generated DCregs

**Figure 4:**
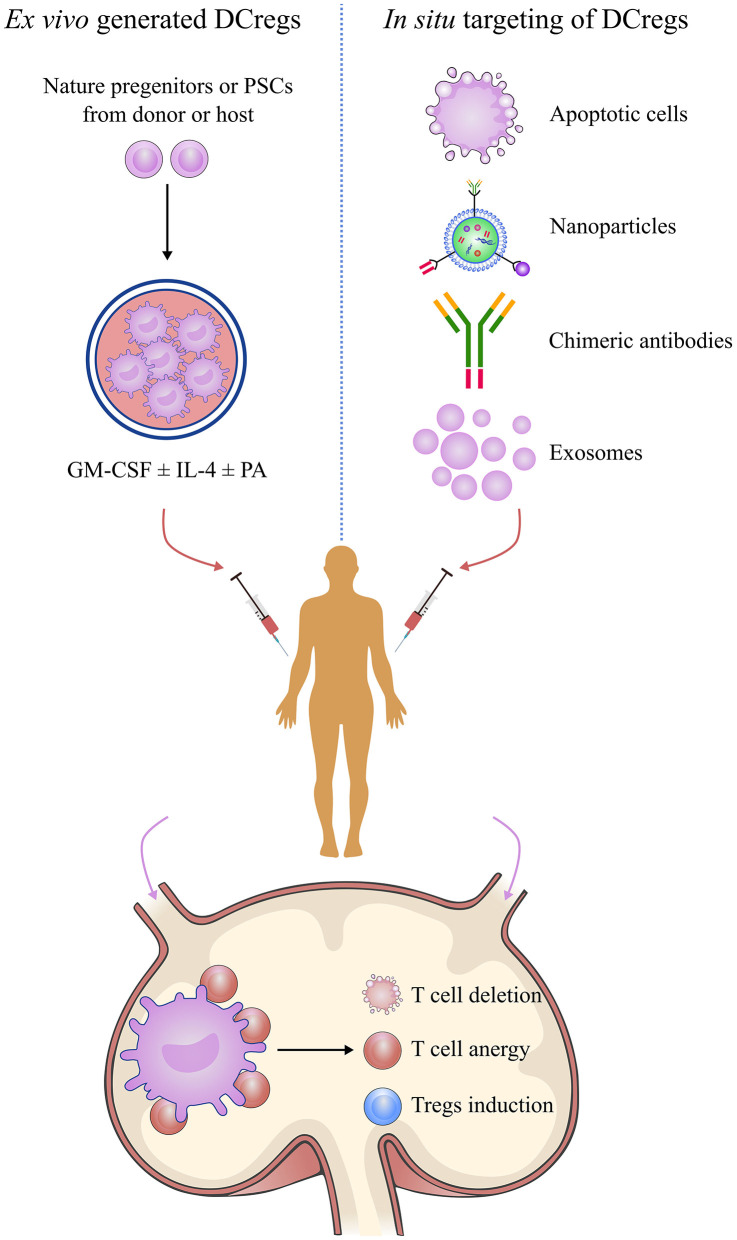
Manipulation of regulatory dendritic cells to promote transplantation tolerance. DCreg-based therapy is a promising strategy for establishing permanent, donor-specific immune tolerance and minimizing or even obviating the use of immunosuppressive drugs in transplantation. Manipulation of DCregs can be done by *in situ* targeting or infusion of *ex vivo*-generated DCregs or mediating donor-specific tolerance via alloreactive T cell anergy, T cell deletion, and Treg induction mechanisms. DCreg, regulatory dendritic cell; PSC, pluripotent stem cell; GM-CSF, granulocyte-macrophage colony-stimulating factor; IL-4, interleukin-4; PA, pharmacological agent; regulatory T cells.

DCreg induction has been intensively studied for its potential therapeutic value in transplantation (Figure 4). The current literature has described various protocols for generating DCregs *ex vivo*. Generally, DCregs are generated from bone marrow precursors in rodents and from CD34+ hematopoietic precursors or blood monocytes (CD14+) in humans (85). The most widely used strategy is the deployment of culture progenitors using granulocyte-macrophage colony-stimulating factor (GM-CSF) ± IL-4 with the addition of one or more pharmacological agents that stably inhibit their maturation and promote their tolerogenicity (86, 87). GM-CSF is a critical cytokine required for *ex vivo* DC generation, but some researchers have used Flt3L instead of GM-CSF for DCreg differentiation (88, 89). The pharmacological agents include IL-10, dexamethasone, vitamin D3, rapamycin, and others (87, 90, 91). DCregs conditioned by IL-10 are resistant to maturation and are capable of inducing antigen-specific T cell anergy and Treg activity (92–94). IL-10-modulated DCs are the most suitable candidate for DC-based transplantation tolerance induction therapy (95). IL-10-induced monocyte-derived DCregs, characterized by a high IL-10/IL-12 ratio and high expression levels of tolerogenic molecules, HLA-G and ILT-4, are potent inducers of adaptive Tr1 cells (96). Dexamethasone-induced DCregs express low levels of costimulatory molecules but high levels of inhibitory receptors, ILT-2 and ILT-3, and produce high amounts of IL-10 and IDO (95, 97, 98). Vitamin D3 and its analogs promote the tolerogenic phenotype of DCreg via the induction of effector T apoptosis and generation of antigen-specific Tregs (90, 99, 100). The combined use of dexamethasone and vitamin D3 leads to “alternatively activated” DCs with an enhanced migration ability inducing memory T cell hyporesponsiveness while skewing naive T cells toward a low IFN-γ/high IL-10 cytokine profile (101, 102). Rapamycin-conditioned DCs have attracted much attention for their role in alloantigen Foxp3+ Treg expansion and migratory activity via enhancing CCR7 expression (91, 103).

Although pharmacological conditioning offers effective strategies for *ex vivo* generation of DCregs, there are some limitations in *ex vivo*-generated DCregs, such as undesirable pro-inflammatory factors, rematuration *in vivo*, and low migratory activity. Genetic manipulation by adenovirus vectors and small interfering RNAs (siRNAs) provides a powerful tool for modifying specific features of *ex vivo*-generated DCregs (104). Previous studies have reported that gene silencing of CD80/CD86 and IL-12 promoted the regulatory activity of DCregs (105, 106). Genetic interference with NF-κB induced a stable immature state by preventing the rematuration of DCregs (107). Dong et al. reported that concurrent CCR7 overexpression and RelB knockdown in DCregs displayed enhanced migratory and regulatory activity (108).

In addition to DCregs generated naturally from progenitor cells, we generated DCregs from murine induced pluripotent stem cells (iPSCs), which were able to remain in a stable immature state even under strong stimulation (109). iPSC-derived DCregs worked as a therapeutic cellular vaccine to generate Tregs and induced donor-specific allograft acceptance. Other researchers have explored using human PSCs, including iPSCs and embryonic stem cells, as a source of DCs for immune therapy (110–112). These studies have highlighted the potential of PSC-derived DCregs in clinical transplantation.

As discussed above, transplantation immunity involves both donor- and host-derived DCs via three allorecognition pathways. Therefore, there is a question of whether DCregs should be derived from the donor or the host. It is also worth noting that the risk of host-sensitization to donor antigens due to the presence of transferred allogeneic molecules should be taken into consideration in DCreg-based therapy. The therapeutic efficacy of DCregs in organ transplantation was first examined by Rastellini et al. and Fu et al. (113, 114). Donor-derived DCregs, infused 1 week before transplantation, significantly prolonged the survival of murine non-vascularized (pancreatic islet) and vascularized (heart) allografts. Alloantigen-specific T-cell anergy was observed *in vitro*. Subsequently, the role of DCreg-based therapy was explored extensively in multiple transplantation models and clinical studies (10). The sources of the DCregs included donor-derived DCregs and host-derived DCregs, either pulsed or unpulsed with donor antigens. Additional host treatment, including costimulation blockade, conventional immunosuppressive drugs, and lymphocyte depletion, was used as an alternative combination to enhance tolerogenic properties and to minimize the risk of host sensitization. These studies have extensively investigated the safety and efficiency of donor- and host-derived DCregs. Although donor-derived DCregs were shown to be capable of inducing transplant tolerance *in vivo*, the underlying mechanisms remain unclear. It is generally assumed that donor-derived DCregs mediate transplantation tolerance via the direct allorecognition pathway, leading to alloreactive T cell anergy, T cell deletion, or Treg induction. However, Divito et al. demonstrated that host DCs mediated the therapeutic effects of the infused donor-derived DCregs (115), which are likely to be rapidly killed by host NK cells or captured and reprocessed by host splenic DCs, which induce Tregs and graft tolerance. This notion was further supported by the finding that the deletion of host DCs abrogates the tolerogenic effects of donor-derived DCregs (116).

Contrary to the accepted paradigm, donor-derived DCregs function as antigen-transporting cells rather than APCs to promote transplantation tolerance. Alternatively, host-derived DCregs, either pulsed or unpulsed with donor antigens, have also been used to regulate the alloreactive T cell response and promote transplantation tolerance (117, 118). Taner et al. showed that infusion with rapamycin-treated, alloantigen-pulsed, host-derived DCregs 1 week before transplantation induced antigen-specific T cell regulation and prolonged graft survival (117). Segovia et al. reported that host-derived DCregs unpulsed with donor antigens, referred to as autologous DCs, when combined with suboptimal doses of immunosuppression, induced prolonged donor-specific allograft survival through tmem176b-dependent antigen cross-presentation (119). Moreover, host-derived DCregs were reportedly more efficient in prolonging cardiac allograft survival than donor-derived DCregs (118, 120). From the clinical perspective, host-derived DCregs appear to be more feasible. Host-derived DCregs can be generated *in vitro* whenever needed while donor-derived DCregs require collecting cells from donors several days prior to transplantation, which is infeasible in cases of deceased organ donation. DCregs using unpulsed, host-derived, immature DCs infused on the day of transplantation decrease the risk of host sensitization against donor antigens (118).

## *In situ* Targeting of DCregs

The infusion of *ex vivo*-generated DCregs in transplantation to regulate the alloreactive T cell response and to promote transplantation tolerance was evident in experimental models and entered clinical studies (10, 121, 122). However, there are some limitations to the clinical application of this approach, including the risk of host sensitization due to the presence of transferred allogeneic molecules; the risk of rematuration after *in vivo* fusion, which could promote alloimmunity rather than tolerance; donor-derived DCregs being unsuitable for deceased organ donation; donor-derived DCregs being quickly eliminated by host NK cells; and the decreased ability to migrate to secondary lymphoid organs to present donor antigens. *In situ* manipulation of DCs is an alternative approach to achieving donor-specific transplantation tolerance. This approach utilizes the *in situ* delivery of immunomodulatory factors targeting DC to regulate the alloreactive T cell response, thereby avoiding the drawbacks of *ex vivo*-generated DCregs, and demonstrates the feasibility of tolerance induction.

The clearance of apoptotic cells by DCs is usually immunologically silent and accompanied by TGF-β and IL-10 release (123). Following the phagocytosis of apoptotic cells, DCs begin to exhibit tolerogenic properties. Donor-derived apoptotic cells have been used to deliver both donor antigens and inhibitory signals simultaneously to host DCs to induce antigen-specific allograft tolerance (124). Engulfing of apoptotic DCs converted immature DCs into DCregs that were resistant to LPS-induced maturation and induced the differentiation of Foxp3+ Treg (125). Exosomes are nano-sized membrane-bound extracellular vesicles produced in the endosomal compartment of most eukaryotic cells (126, 127). The phenotype and function of exosomes depend on the origin and state of the cell. DC-derived exosomes may bear MHC molecules, costimulatory molecules, and antigens and function as antigen-presenting nanovesicles (128, 129). Recent research has revealed the role of DC-derived exosomes in allorecognition and transplantation immunity (130). The tolerogenic function of DC-derived exosomes was demonstrated in experimental transplantation models (131, 132). Exosomes from immature, donor-derived DCs induced donor-specific allograft tolerance. Host DCs engulfed these exosomes and presented intact, donor MHC antigens (allo-MHC cross-dressing) and immunomodulatory molecules, such as IL-10, PD-L1, and IDO, in what was considered to be the mechanism underlying microchimerism and tolerance induction (130, 133).

Nanoparticle-based drug delivery systems are a valuable tool in modulating DCs *in situ* by enabling the direct delivery of encapsulated antigens and immunomodulatory agents via cell-specific targeting *in vivo*, thus facilitating precise immune regulation to induce transplantation tolerance (134). The poly (lactic-co-glycolic acid) (PLGA) nanoparticles approved by The Food and Drug Administration are the most frequently used nanocarriers. Nanoparticles can also be engineered by coating monoclonal antibodies so that they target specific DC subsets (135, 136). Delivery to DCs can be achieved by targeting DC receptors, including CD11c, CD40, CD205, CD206, CD209, and Fc receptors. The encapsulated agents are protected from enzymatic and chemical degradation, act directly on the DCs, and are more efficient than methods of systemic administration, especially of toxic reagents. Nanoparticle delivery of mycophenolic acid upregulated PD-L1 expression on DCs, prolonged mouse skin allograft survival, and avoided the toxicity of soluble drug delivery (137). Maldonado et al. induced antigen-specific immunological tolerance using polymeric synthetic nanoparticles loaded with antigens and rapamycin, which resulted in suppression of T cell activation and an increase in regulatory cells (138). Clustered regularly interspaced short palindromic repeat (CRISPR)/CRISPR-associated protein 9 (Cas9) is emerging as a powerful tool for engineering the genome in diverse organisms. Zhang et al. encapsulated Cas9 mRNA (mCas9) and a guide RNA targeting CD40 (gCD40) with nanoparticles (139). mCas9/gCD40 was effectively delivered into DCs and disrupted CD40 signaling, significantly protecting grafts from acute rejection-mediated injury and prolonging graft survival. We also developed a novel siRNA delivery system with a poly-dA extension at the 5′-end of the siRNA sense strand that was stably incorporated into 1,3-β-glucan (schizophyllan, SPG) (140). siRNAs silencing the CD40 gene were delivered into DCs through its receptor, Dectin-1, resulting in antigen-specific Treg generation and permanent murine cardiac allograft tolerance. Moreover, nanoparticles have also been used as integrating imaging moieties for monitoring allograft rejection to provide diagnostic and prognostic information as well as for quantifying the treatment efficacy in transplant recipients (134).

Antigen delivery to DCs *in vivo* using recombinant chimeric antibodies, produced by genetically modifying original monoclonal antibodies and chemically coupling them with peptide antigens, is another promising approach to achieving specific immunomodulation (141, 142). This approach enables the delivery of antigens to DCs under steady-state conditions and the induction of peripheral tolerance (143, 144). Reeves et al. have shown that APC-targeted proinsulin expression converted insulin-specific CD8+ T-cell priming to tolerance in autoimmune-prone NOD mice (145). Ettinger et al. achieved prolonged survival of transgenic mouse skin grafts by utilizing an antibody recognizing the CD205 receptor to deliver the immunodominant domain of type XVII collagen to host DCs without inflammatory stimuli (146).

## DCregs in Clinical Organ Transplantation

In rodent and non-human primate transplantation studies, DCregs exhibited promising potential as a natural, well-tolerated, antigen-specific therapeutic strategy capable of promoting lasting transplantation tolerance. The clinical study of the safety and efficacy of DCregs in transplantations has lagged behind that of autoimmune diseases, including type-1 diabetes, rheumatoid arthritis, and Crohn's disease, which have reported early safety data (147). Recently, early-phase clinical trials of DCregs in living-donor renal and liver transplantations have begun both in Europe and the US (10, 148).

At the University of Nantes, investigators launched a phase I/II trial of unpulsed autologous DCregs in living-donor renal transplantation (clinicaltrials.gov identifier: NCT0225055). The autologous, monocyte-derived DCregs were generated in a low concentration of GM-CSF and infused into hosts 1 day before transplantation. The hosts also received background immunosuppression with prednisolone, mycophenolate mofetil, and tacrolimus. Recently, the first results and related good manufacturing practice protocols have been published (149, 150). At the University of Pittsburgh, a three-arm, dose-escalation, phase I clinical trial evaluating the safety and feasibility of donor-derived DCreg using a single infusion 1 week prior to living-donor renal transplantation, in combination with mycophenolate mofetil steroid and tacrolimus immunosuppression therapy, is currently underway (NCT0364265).

Given the unique immunological function of the liver, almost half of highly selected liver transplant recipients showed good tolerance even after complete weaning from immunosuppression (151, 152). DCreg-based therapy contributing to antigen-specific tolerance induction may facilitate minimizing immunosuppression and early weaning after liver transplantation. At the University of Pittsburgh, Angus et al. has initiated two phase I/II trials to investigate the safety and efficacy of a single infusion of donor-derived DCreg 1 week before transplantation (NCT03164265) and 1 week before immunosuppression weaning (NCT04208919), respectively, in living donor liver transplant recipients. The donor monocyte-derived DCregs were generated in the presence of IL-10 and Vitamin D3 (153), and the hosts were slowly weaned off immunosuppression after meeting specific criteria.

## Concluding Remarks

Dendritic cells are central instigators and regulators of transplantation immunity and are critical in the balance between allograft rejection and tolerance. Extensive studies focusing on the development, phenotype, and function of DCs have provided important insights into the mechanisms underlying tolerance induction. DCregs comprise a heterogeneous population of immature or semi-mature DCs which expressed low levels of MHC, costimulatory molecules, and altered cytokine production and mediated donor-specific tolerance through alloreactive T cell anergy, T cell deletion, and Treg induction. DCreg-based therapy, by *in situ* targeting or infusion of *ex vivo*-generated DCregs represents an emerging approach to preventing rejection and promoting donor-specific tolerance. Further studies are required to explore the translation of DCregs into clinical transplantation to induce tolerance and improve allograft acceptance.

## Author Contributions

WQ and W-ZG did the literature search and wrote the review. X-KL revised and approved the final version of the review. All authors contributed to the article and approved the submitted version.

## Conflict of Interest

The authors declare that the research was conducted in the absence of any commercial or financial relationships that could be construed as a potential conflict of interest.
